# CRISPR/Cas9-Targeted Mutagenesis of *BnaFAE1* Genes Confers Low-Erucic Acid in *Brassica napus*

**DOI:** 10.3389/fpls.2022.848723

**Published:** 2022-02-10

**Authors:** Yunhao Liu, Zhuolin Du, Shengli Lin, Haoming Li, Shaoping Lu, Liang Guo, Shan Tang

**Affiliations:** ^1^National Key Laboratory of Crop Genetic Improvement, Huazhong Agricultural University, Wuhan, China; ^2^Hubei Hongshan Laboratory, Wuhan, China

**Keywords:** *Brassica napus*, CRISPR/Cas9, *FAE1*, erucic acid, seed oil content

## Abstract

Rapeseed (*Brassica napus*) is an important oilseed crop widely planted in the world, providing substantial edible oil and other nutrients for mankind. The composition of fatty acids affects the edible and processing quality of vegetable oils, among which erucic acid (EA) is potentially to cause health problems. Therefore, low erucic acid (LEA) has always been a breeding trait of *B. napus*. *Fatty acid elongase 1* (*FAE1*) plays a decisive role in the synthesis of EA. There are two functional homologous copies of *FAE1* on the A08 and C03 chromosomes in *B. napus*. In this study, we used CRISPR/Cas9 technology to create targeted mutations on these two homologous copies of *BnaFAE1* in three *B. napus* germplasms with high EA (>30%) and high oil (>50%). Our results show that the EA content was significantly reduced by more than 10 percentage points in the mutant of *BnaC03.FAE1* (*c03*), while the double mutation of *BnaA08.FAE1* and *BnaC03.FAE1* (*a08c03*) resulted in nearly zero EA in three *BnaFAE1-*edited germplasms, and the oleic acid content was increased in different degrees. In addition, knockout of *BnaA08.FAE1* or/and *BnaC03.FAE1* mildly decreased seed oil content, but had no significant effect on other agronomic traits. In general, we successfully created low EA germplasms of *B. napus*, which provides a feasible way for future low EA breeding.

## Introduction

In oilseeds, the *de novo* synthesis of fatty acids occurs in plastids with acetyl-coenzyme A (CoA) as substrates. Acetyl-CoA carboxylase (ACC) catalyzes the condensation of acetyl-CoA and CO_2_ to form malonyl-CoA, then the malonyl group of malonyl-CoA is transferred from CoA to acyl carrier protein (ACP) under the catalysis of malonyl-CoA:acyl carrier protein malonyltransferase (MCMT). Acetyl-CoA and malonyl-ACP, respectively, enter the fatty acid synthesis complex (FAS) and undergo a series of reactions including condensation, reduction, dehydration, and re-reduction to form C4:0-ACP. Going through the same cycle reaction, C4:0-ACP reacts with acetyl-CoA, adding two carbons every cycle, to produce C16:0-ACP eventually. In the first cycle, the condensation reaction is catalyzed by ketoacyl-ACP synthase III (KAS III) and the condensation reactions in the next six turns of the cycles are then catalyzed by ketoacyl-ACP synthase I (KAS I). Each cycle uses malonyl-ACP as a source of 2C units (Ohlrogge and Browse, 1995; Li-Beisson et al., 2013). The synthesized C16:0-ACP is extended to C18:0-ACP under the action of 3-ketoacyl-ACP synthases II (KAS II), then C18:0-ACP is desaturated to form C18:1-ACP by stearoyl-ACP desaturase (SAD). The synthesized C16- or C18-ACP are released from FAS to form free fatty acids by acyl-ACP thioeserase (FAT), and the fatty acids are catalyzed to acyl-CoA by long-chain acyl-CoA synthase (LACS) (Chapman and Ohlrogge, 2012; Li-Beisson et al., 2013). These acyl-CoA are transported to the endoplasmic reticulum, and then the fatty acid chain is desaturated and extended. Oleic acid (C18:1) is desaturated to form linoleic acid (C18:2) and linolenic acid (C18:3) under the catalysis of fatty acid desaturase 2 (FAD2) and fatty acid desaturase 3 (FAD3), or is extended to C20-C24 very long-chain fatty acids (VLCFAs) by the fatty acid elongase 1 (*FAE1*) (Browse and Somerville, 1991; Li-Beisson et al., 2013).

Emergence of CRISPR-Cas9 provides researchers and breeders a powerful tool to study gene function and obtain desired traits by precise and efficient mutagenesis of specific genes (Razzaq et al., 2019; Li et al., 2021; Zhang et al., 2021). In recent years, gene editing technology has been widely used in fatty acid improvement. CRISPR-Cas9 mediated genome editing of *FAD2* could produce high oleic acid/low linoleic acid seeds in *Camelina sativa* (Jiang et al., 2017; Morineau et al., 2017), rice (Abe et al., 2018; Bahariah et al., 2021), rapeseed (Okuzaki et al., 2018; Huang et al., 2020), peanut (Yuan et al., 2019), soybean (Pham et al., 2012; Al Amin et al., 2019; Wu et al., 2020), which provides a new idea for the breeding of oil crops with high oleic acid. In addition, knocking out *FAE1* by CRISPR technology could significantly reduce VLCFAs from 22 to <2% in *C. sativa* (Ozseyhan et al., 2018).

Rapeseed is one of the most important oil crops and produces ^~^13% of edible oil globally (Tang et al., 2021). Erucic acid (EA, *cis*-D13 C22:1 fatty acid, hereafter abbreviated as C22:1) is found in many vegetable oils. It has been publicly recognized that EA is one of the major factors that restrain the utilization of rapeseed oil containing high EA for edible oil (Knutsen et al., 2016). In the history of rapeseed genetic improvement, low erucic acid (LEA) revolution made great contributions to the popularization of rapeseed oil. In 1960s, a natural LEA mutant was identified in a feed rapeseed “Liho” (Stefansson et al., 1961). F1 seeds from cross between this mutant and a high EA variety displayed intermediate EAC between those of its parents, suggesting the genetic regulators of EAC act in an additive manner. The segregation ratios of EACs in F2 and F3 seeds were in good agreement with the theoretical ratios under regulation of two genes (Harvey and Downey, 1964). These two genes were identified to be *BnaFAE1* in rapeseed and two *BnaFAE1* genes on chromosome A08 and C03 play major roles in the synthesis of EA (Gupta et al., 2004; Qu et al., 2017).

At present, breeders own multiple *Brassica napus* germplasms with high seed oil content (SOC), but they cannot be well utilized in breeding because many high SOC germplasms contain high EAC. In order to improve the EAC of three germplasms and evaluate the impact of *BnaFAE1* on the agronomic traits of *B. napus*, we used gene editing technology to knock out the *BnaFAE1* genes, and finally obtained *BnaFAE1* knockout mutants with reduced EAC. The EAC of *BnaA08.FAE1* and *BnaC03.FAE1* double mutants were almost reduced to zero, while the content of C18:1 was greatly increased to more than 66%. This study provides new LEA germplasm resources for the breeding of *B. napus*.

## Materials and Methods

### Plant Materials

The germplasms used in this study were three high SOC and high EA *B. napus* inbred lines WH3411, WH3417, and GY284, which were obtained from the National Engineering Research Center of Rapeseed, Wuhan, China.

### Sequence Alignment and Gene Expression Analysis

Amino acid sequences in this research were found from the Tair^1^ and *B. napus* transcriptome information resource (BnTIR)^2^ (Liu et al., 2021). Amino acid sequence alignment was performed by MEGA7 and gene expression data of *FAE1*s in *B. napus* were obtained from BnTIR (Liu et al., 2021).

### Construction of CRISPR/Cas9 Vector

To generate *BnaFAE1* mutants, two sgRNAs simultaneously targeting at *BnaA08.FAE1* and *BnaC03.FAE1* were designed by CRISPR-P^3^ (Lei et al., 2014) and putative off-target sites were manually eliminated. U6-26 and U6-29 promoters from Arabidopsis were employed to separately drive these two sgRNA cassettes, which were fused in T-DNA region of pKSE410 vector carrying a Kanamycin selection marker (Xing et al., 2014). Primers used in the construction of the CRISPR/Cas9 vector were listed in Supplementary Table 1.

### *Agrobacterium*-Mediated Transformation of *Brassica napus*

*Agrobacterium tumefaciens* (GV3101 strain) cells were transfected with the BnaFAE1-CRISPR-Cas9 recombinant plasmid by electroporation method. *A. tumefaciens*-mediated hypocotyl transformation in *B. napus* were conducted as previously described (Dai et al., 2020).

### Identification of *BnaFAE1* Mutants

T0 plants were obtained by kanamycin screening (25 mg/L), and the Cas9 protein was identified by primer pairs Cas9F/R. Then the positive plants with Cas9 were selected to amplify *BnaA08.FAE1* and *BnaC03.FAE1*, respectively, and the amplified fragments were sequenced and analyzed to identify edited T0 mutants. To obtain homozygous mutants, the T0 mutants were self-crossed for T1 and T2 generations and confirmed by sequencing. Primers used in the identification were listed in Supplementary Table 1.

### Field Experiments and Investigation of Agronomic Traits

T0 and T1 mutant plants and WT plants were grown in a greenhouse (16/8 h of light/dark at 22°C) in 2018 and 2019, respectively. The confirmed homozygous T2 mutant lines without Cas9 were grown in the winter-type growing season (2020–2021) in the experimental farm of Huazhong Agricultural University, Wuhan, China. The field experiment followed a randomized complete block with three replications. Each line was planted in one row with 8–10 plants, with a distance of 21 cm between plants within each row and 30 cm between rows. The field management was performed in line with standard breeding practice. Yield-related traits including plant height, branch height, branch number, silique length, number of siliques per plant, 1000-seed weight, and yield per plant were measured as described previously (Cai et al., 2016).

### Analysis of Seed Quality-Related Traits

Mature seeds were harvested and dried for the measurement of seed quality-related traits, including fatty acids composition and SOC. Fatty acids were extracted using the gas chromatograph (GC) fatty acid methyl ester method as described previously (Lu et al., 2016). A total of nine fatty acid species were measured with an Agilent 6890 GC. SOC is scanned by near infrared spectroscopy using 2000–3000 seeds per scan (Gan et al., 2003).

## Results

### Selection and Identification of Three High Erucic Acid and High Seed Oil Content *Brassica napus* Seeds

**FIGURE 1:**
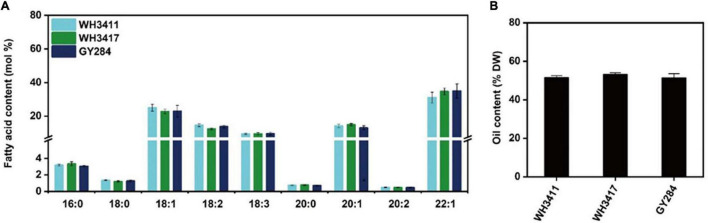
Fatty acid composition and oil content of three germplasms (WH3411, WH3417, GY284). **(A)** Fatty acids were extracted from mature seeds and analyzed using the gas chromatograph method. Values are means ± SD (*n* = 3∼5). **(B)** Seed oil content is determined by near infrared spectroscopy. Values are means ± SD (*n* = 12∼20).

Three natural *B. napus* germplasms WH3411, WH3417, and GY284 were selected and their fatty acid composition characters were measured. Fatty acids were determined by GC analysis, and the results show that EA of these three germplasms were between 31.05 and 34.95 mol% (Figure 1A). SOC was measured by near infrared spectroscopy, and the SOC of three germplasms ranged from 51.28 to 53.08% (Figure 1B). The results show that WH3411, WH3417, and GY284 have high EAC and high SOC.

### Creation of *BnaFAE1* Mutants by CRISPR/Cas9

**FIGURE 2:**
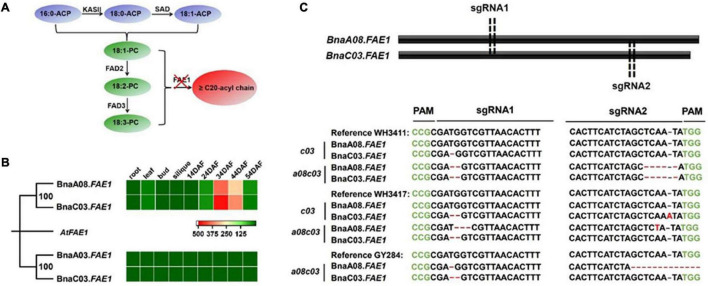
*BnaFAE1* gene analysis and mutant generation. **(A)** Illustration of desaturation and elongation of fatty acids. Red cross indicates mutation of *FAE1* genes to block the synthesis of EA. **(B)** Expression pattern of *BnaFAE1s* in different tissues. **(C)** Location of CRISPR/Cas9 sgRNA-1 and sgRNA-2 targeting *BnaFAE1* genes and sequencing identification of T2 homozygous mutants. PAM is indicated in green. Red “-” means deletions. Red font indicates nucleotide insertions and substitutions.

In order to reduce EA in above three germplasms, CRISPR/Cas9 technology was employed to knock out *BnaFAE1s* (Figure 2A). There are four homologous copies of *BnaFAE1* in *B. napus* and the expression data in different tissues showed that *BnaA03.FAE1* and *BnaC03.FAE1-2* were barely expressed in different tissues, while *BnaA08.FAE1* and *BnaC03.FAE1* were mainly expressed in the developing seeds, especially in the middle and late periods of seed development (Figure 2B). Based on the expression levels, *BnaA08.FAE1* and *BnaC03.FAE1* were selected to design target mutation sites. Both *BnaA08.FAE1* and *BnaC03.FAE1* were about 1500 bp in size and only consisted of one exon. We designed target sites at ^~^600 and 1300 bp, respectively. As a result, homozygous *BnaC03.FAE1* mutations (*c03*) of WH3411, WH3417, and GY284, and homozygous *BnaA08.FAE1* and *BnaC03.FAE1* double mutations (*a08c03*) of WH3411 and WH3417 were identified by sequencing in T2 generation (Figure 2C and Supplementary Figure 1). All of them cause early termination of translation except *a08c03^WH3417^* has one amino acid deletion and one amino acid mutations in the *BnaA08.FAE1* (Supplementary Figure 2).

### CRISPR/Cas9-Induced Mutations in *BnaFAE1s* Greatly Reduce Erucic Acid Content in *Brassica napus* Seed

**FIGURE 3:**
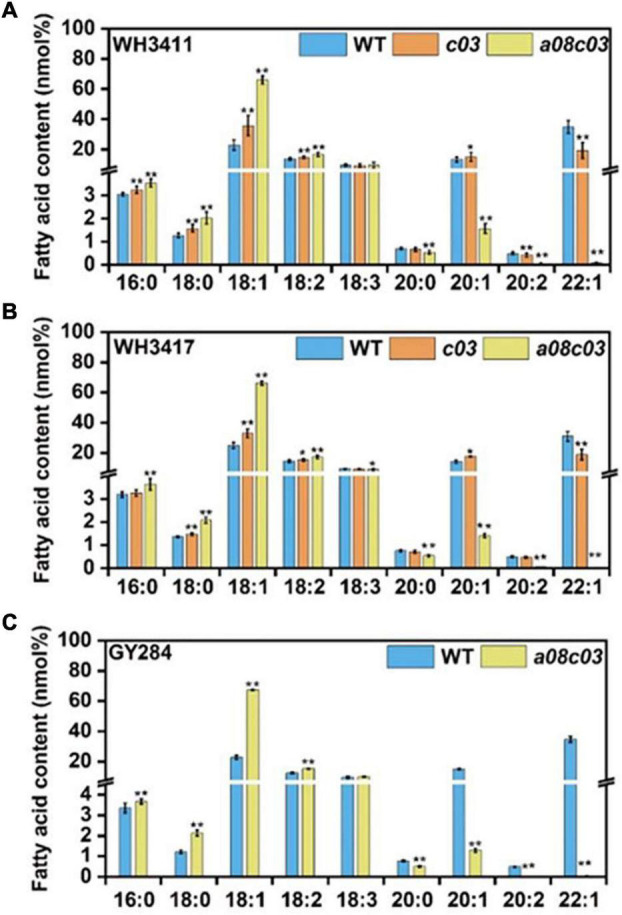
The fatty acid composition phenotype of T2 mutants. Phenotype of fatty acid composition in WH3411 **(A)**, WH3417 **(B)**, and GY284 **(C)** backgrounds. *c03* represents *BnaC03.FAE1* homozygous mutant. *a08c03* represents *BnaC03.FAE1* and *BnaA08.FAE1* homozygous double mutant. Values are means ± SD (*n* = 3∼5). *P ≤ 0.05; **P ≤ 0.01.

We analyzed the fatty acids of mature seeds of T2 generation by GC method, and the results showed that the C22:1 of *c03* and *a08c03* was decreased from 34.9 to 19.3 and 0.07% when WH3411 was used as receptor. In addition, the composition of oleic acid (C18:1) in *c03* and *a08c03* was increased from 22.9 to 35.6 and 66.0%, respectively. Moreover, the composition of linoleic acid (C18:2) was increased to varied degrees (Figure 3A). In WH3417, the C22:1 of *c03* and *a08c03* was decreased from 31.0 to 18.8 and 0.03%, respectively. Meanwhile, C18:1 was increased from 25.0 to 32.9 and 66.2% in *c03* and *a08c03*, respectively (Figure 3B). Only homozygous *a08c03* double mutant was obtained in GY284 background. The composition of C22:1 was reduced from 34.6 to 0.02%. C18:1 was increased from 22.8 to 67.3% and C18:2 was increased from 12.4 to 15.2% (Figure 3C). These results suggest that knocking out of *BnaFAE1s* can greatly reduce EAC and increase the content of oleic acid and linoleic acid in *B. napus*.

### Mutation of *BnaFAE1* Results in Mild Decrease of Seed Oil Content

**FIGURE 4:**
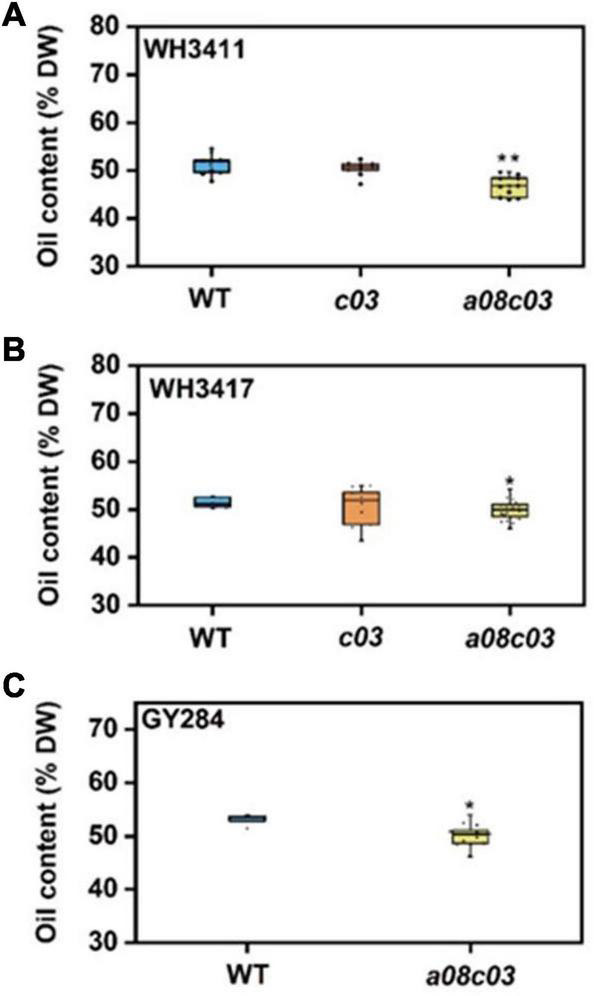
Seed oil content of *BnaFAE1* mutants. Seed oil content of WH3411 **(A)**, WH3417 **(B)**, and GY284 **(C)** and their Bna*FAE1* mutants was determined by near infrared spectroscopy. *c03* represents *BnaC03.FAE1* homozygous mutant. *a08c03* represents *BnaC03.FAE1* and *BnaA08.FAE1* homozygous double mutant. Values are means ± SD (*n* = 6∼20). *P ≤ 0.05; **P ≤ 0.01.

To determine whether the mutation of *BnaFAE1* affects the SOC, SOC of these mutant lines was analyzed by near infrared spectroscopy. The results indicate that the SOC of *BnaC03.FAE1* mutant (*c03*) was not significantly altered in WH3411 and WH3417 background (Figures 4A,B). The SOC of *BnaA08.FAE1* and *BnaC03.FAE1* double mutants (*a08c03*) was significantly reduced from 51.28, 51.49, and 53.08% to 46.69, 49.96, and 50.17%, respectively, in WH3411, WH3417, and GY284 background (Figures 4A–C). The results indicate that knocking out of *BnaA08.FAE1* and *BnaC03.FAE1* simultaneously could slightly reduce seed oil accumulation in *B. napus*.

### Investigation of Agronomic Trait in Field

**FIGURE 5:**
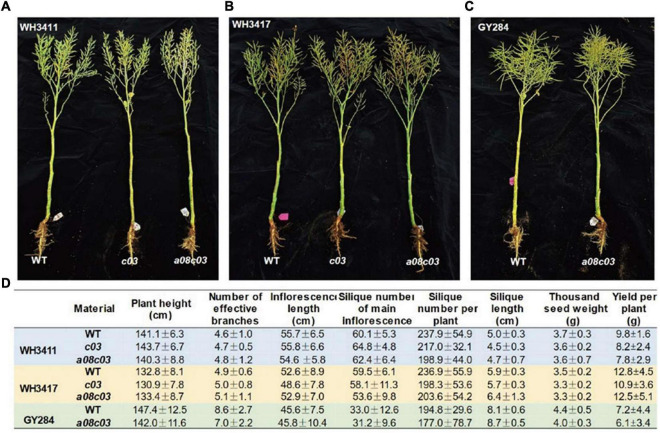
Agronomic traits investigation. **(A–C)** Morphology of WH3411, WH3417, GY284, and their Bna*FAE1* mutants. **(D)** Comparison of agronomic traits of WH3411, WH3417, GY284 with their *FAE1* mutants. Values are means ± SD (*n* = 6∼8).

To evaluate the impact of knockout of *BnaFAE1s* on the agronomic traits, mutant lines were sown in field under the natural environment. During the whole growth period, the mutants did not show obvious visible difference in growth. At mature stage, these mutants did not exhibit obvious morphological changes compared with WT (Figures 5A–C). Meantime, we investigated the agronomic traits including plant height, branch number, branch length, silique number, silique length, thousand seed weight, and yield. The results show that these agronomic traits were not significantly altered in these mutants (Figure 5D), indicating that knockout of *BnaA08.FAE1* or/and *BnaC03.FAE1* had no significant effect on plant growth and yield.

## Discussion

The synthetic pathway of EA involves a variety of enzymes, including 3-ketoacyl-CoA synthase (KCS), 3-ketoacyl-CoA reductase (KCR), 3-hydroxyacyl-CoA dehydratase (HCD), and *trans*-2,3-enoyl-CoA reductase (ECR) (Yu et al., 2011). Among these, the KCS encoded by *FAE1* was the most critical one (Millar and Kunst, 1997). Therefore, finding or creating *BnaFAE1* mutants has become an important way to cultivate LEA *B. napus* varieties. Until now, there are two main methods to acquire LEA mutants. One is to screen from natural or EMS mutagenic mutants (Harvey and Downey, 1964; Wang et al., 2008), and the other is to inhibit *BnaFAE1* expression by RNAi (Js et al., 2017). In this study, new LEA mutants were created using CRISPR/Cas9-driven knockout of *BnaFAE1* using three high SOC and high EAC germplasms, which broadens the breeding resources of *B. napus* with LEA.

In addition, this study shows that CRISPR/Cas9 induced mutation of the *BnaFAE1* genes significantly changed the fatty acid profiles in seeds, resulting in significantly decreased EA. The reduction of EA in the double mutant (*a08c03*) is much stronger than that in the single mutant (*c03*), which indicates that *BnaA08.FAE1* and *BnaC03.FAE1* have a dose effect on EA level, and *BnaA08.FAE1* and *BnaC03.FAE1* have certain functional redundancy. This is also consistent with previous results (Stefansson and Hougen, 1964). Previous studies have reported that the content of VLCFAs in the *FAE1* mutants of *Arabidopsis* was greatly reduced, while the content of oleic acid was significantly increased (Lemieux et al., 1990). In this study, we also observed similar results, especially in the *BnaA08.FAE1* and *BnaC03.FAE1* double mutants (*a08c03*), and the oleic acid content significantly increased (over 66%), accompanying with the increase of linoleic acid (Figure 3). Taken together, our results demonstrate that knockout of the *BnaFAE1s* substantially improves the nutritional quality of *B. napus* seed oil.

Owing to the significance of high SOC and LEA in production, understanding of fatty acid metabolism and seed oil accumulation has obvious practical application value in oil crop breeding. Previous studies showed that *BnaFAE1* was significantly associated with SOC (Li et al., 2014). Ecke et al. (1995) used the double haploid (DH) population to locate three SOC QTLs in the rape genome, and found that two of them were highly correlated with *BnaA08.FAE1* and *BnaC03.FAE1*, and each additional high EA allele increased the SOC by 1 percentage point. Our results show that when *BnaC03.FAE1* was knocked out, the SOC was not significantly decreased, and when both *BnaA08.FAE1* and *BnaC03.FAE1* were knocked out, the SOC was decreased by 1.53–4.59%. This is consistent with previous findings that inhibition of *BnaFAE1* expression significantly reduces the SOC (Js et al., 2017). In order to make up this penalty on oil content, favorable genes/alleles such as DAGT may be introduced into the mutant (*a08c03*) to promote seed oil accumulation (Taylor et al., 2009). Both *BnaA08.FAE1* and *BnaC03.FAE1* are highly expressed in developing seeds while have low expression in other tissues. It is not surprising that knockout of *BnaA08.FAE1* or/and *BnaC03.FAE1* had no obvious effect on agronomic traits and plant architecture of *B. napus*. Above results suggest that it is feasible to breed LEA *B. napus* using high EA germplasms by direct genome editing of *BnaA08.FAE1* and *BnaC03.FAE1*.

## Conclusion

In brief, this is the first report using CRISPR/Cas9 to create LEA germplasms of *B. napus* by mutating *BnaFAE1s* in three germplasms with consistent results. The EAC was significantly reduced when *BnaA08.FAE1* or/and *BnaC03.FAE1* were mutated in different germplasms. The EA content was reduced to nearly zero when *BnaA08.FAE1* and *BnaC03.FAE1* were both knocked out. Our findings reveal that knockout of *BnaA08.FAE1* or/and *BnaC03.FAE1* had no remarkable effects on agronomic traits except mildly decreased SOC. Our work successfully generated new LEA germplasms for breeding LEA *B. napus*.

## Data Availability Statement

The original contributions presented in the study are included in the article/Supplementary Material, further inquiries can be directed to the corresponding authors.

## Author Contributions

LG and ST designed this study. ZD, YL, SLi, and HL performed the experiments. YL and ST analyzed the data and wrote the manuscript. LG, ST, and SLu revised the manuscript. All authors contributed to the article and approved the submitted version.

## Conflict of Interest

The authors declare that the research was conducted in the absence of any commercial or financial relationships that could be construed as a potential conflict of interest.

## Publisher’s Note

All claims expressed in this article are solely those of the authors and do not necessarily represent those of their affiliated organizations, or those of the publisher, the editors and the reviewers. Any product that may be evaluated in this article, or claim that may be made by its manufacturer, is not guaranteed or endorsed by the publisher.

## Footnotes

1
https://www.arabidopsis.org/

2
http://yanglab.hzau.edu.cn/BnTIR

3
http://crispr.hzau.edu.cn/CRISPR/

## References

[B1] Abe K. Araki E. Suzuki Y. Toki S. Saika H. (2018). Production of high oleic/low linoleic rice by genome editing. *Plant Physiol. Biochem.* 131 58–62. 10.1016/j.plaphy.2018.04.033 29735369

[B2] Al Amin N. Ahmad N. Wu N. Pu X. Ma T. Du Y. (2019). CRISPR-Cas9 mediated targeted disruption of FAD2-2 microsomal omega-6 desaturase in soybean (*Glycine max*. L). *BMC Biotechnol.* 19:9. 10.1186/s12896-019-0501-2 30691438 PMC6350355

[B3] Bahariah B. Masani M. Y. A. Rasid O. A. Parveez G. K. A. (2021). Multiplex CRISPR/Cas9-mediated genome editing of the FAD2 gene in rice: a model genome editing system for oil palm. *J. Genet. Eng. Biotechnol.* 19:86. 10.1186/s43141-021-00185-4 34115267 PMC8196110

[B4] Browse J. Somerville C. (1991). Glycerolipid synthesis: biochemistry and regulation. *Annu. Rev. Plant Physiol. Plant Mol. Biol.* 42 467–506. 10.1146/annurev.pp.42.060191.002343

[B5] Cai G. Yang Q. Chen H. Yang Q. Zhang C. Fan C. (2016). Genetic dissection of plant architecture and yield-related traits in *Brassica napus*. *Sci. Rep.* 6:21625. 26880301 10.1038/srep21625 PMC4754947

[B6] Chapman K. D. Ohlrogge J. B. (2012). Compartmentation of triacylglycerol accumulation in plants. *J. Biol. Chem.* 287 2288–2294. 10.1074/jbc.R111.290072 22090025 PMC3268389

[B7] Dai C. Li Y. Li L. Du Z. Lu S. (2020). An efficient Agrobacterium-mediated transformation method using hypocotyl as explants for *Brassica napus*. *Mol. Breed.* 40:96. 10.1007/s11032-020-01174-0

[B8] Ecke W. Uzunova M. Weileder K. (1995). Mapping the genome of rapeseed (*Brassica napus* L.). II. Localization of genes controlling erucic acid synthesis and seed oil content. *Theor. Appl. Genet.* 91 972–977. 10.1007/BF00223908 24169985

[B9] Gan L. Sun X. L. Jin L. Wang G. Xiu J. Wei Z. (2003). Establishment of math models of NIRS analysis for oil and protein contents in seed of *Brassica napus*. *Sci. Agric. Sin.* 36 1609–1613.

[B10] Gupta V. Mukhopadhyay A. Arumugam N. Sodhi Y. S. Pental D. Pradhan A. K. (2004). Molecular tagging of erucic acid trait in oilseed mustard (*Brassica juncea*) by QTL mapping and single nucleotide polymorphisms in FAE1 gene. *Theor. Appl. Genet.* 108 743–749. 10.1007/s00122-003-1481-z 14564400

[B11] Harvey B. L. Downey R. K. (1964). The inheritance of erucic acid content in rapeseed (*Brassica napus*). *Can. J. Plant Sci.* 44 104–111. 10.4141/cjps64-019

[B12] Huang H. Cui T. Zhang L. Yang Q. Yang Y. Xie K. (2020). Modifications of fatty acid profile through targeted mutation at BnaFAD2 gene with CRISPR/Cas9-mediated gene editing in *Brassica napus*. *Theor. Appl. Genet.* 133 2401–2411. 10.1007/s00122-020-03607-y 32448919

[B13] Jiang W. Z. Henry I. M. Lynagh P. G. Comai L. Cahoon E. B. Weeks D. P. (2017). Significant enhancement of fatty acid composition in seeds of the allohexaploid, *Camelina sativa*, using CRISPR/Cas9 gene editing. *Plant Biotechnol. J.* 15 648–657. 10.1111/pbi.12663 27862889 PMC5399004

[B14] Js A. Cl A. Fw A. Xw A. Rl A. Tao Z. A. (2017). Depressed expression of FAE1 and FAD2 genes modifies fatty acid profiles and storage compounds accumulation in *Brassica napus* seeds. *Plant Sci.* 263 177–182. 10.1016/j.plantsci.2017.07.014 28818373

[B15] Knutsen H. K. Alexander J. Barregård L. Bignami M. Brüschweiler B. Ceccatelli S. (2016). Erucic acid in feed and food. *EFSA J.* 14:e04593. 10.2903/j.efsa.2016.4593

[B16] Lei Y. Lu L. Liu H. Y. Li S. Xing F. Chen L. L. (2014). CRISPR-P: a web tool for synthetic single-guide RNA design of CRISPR-system in plants. *Mol. Plant* 7 1494–1496. 10.1093/mp/ssu044 24719468

[B17] Lemieux B. M. M. Miquel M. Somerville C. R. Browse J. (1990). Mutants of Arabidopsis with alterations in seed lipid fatty-acid composition. *Theor. Appl. Genet.* 80 234–240. 10.1007/BF00224392 24220901

[B18] Li C. Brant E. Budak H. Zhang B. (2021). CRISPR/Cas: a Nobel Prize award-winning precise genome editing technology for gene therapy and crop improvement. *J. Zhejiang Univ. Sci. B.* 22 253–284. 10.1631/jzus.B2100009 33835761 PMC8042526

[B19] Li F. Chen B. Xu K. Wu J. Song W. Ian B. (2014). Genome-wide association study dissects the genetic architecture of seed weight and seed quality in rapeseed (*Brassica napus* L.). *DNA Res.* 21 355–367. 10.1093/dnares/dsu002 24510440 PMC4131830

[B20] Li-Beisson Y. Shorrosh B. Beisson F. Andersson M. X. Arondel V. Bates P. D. (2013). Acyl-lipid metabolism. *Arabidopsis Book* 11:e0161. 10.1199/tab.0133 23505340 PMC3563272

[B21] Liu D. Yu L. Wei L. Yu P. Wang J. Zhao H. (2021). BnTIR: an online transcriptome platform for exploring RNA-seq libraries for oil crop *Brassica napus*. *Plant Biotechnol. J.* 19 1895–1897. 10.1111/pbi.13665 34260132 PMC8486221

[B22] Lu S. Yao S. Wang G. Guo L. Zhou Y. Hong Y. (2016). Phospholipase Dε enhances *Brassica napus* growth and seed production in response to nitrogen availability. *Plant Biotechnol. J.* 14 926–937. 10.1111/pbi.12446 26260942 PMC11388816

[B23] Millar A. A. Kunst L. (1997). Very-long-chain fatty acid biosynthesis is controlled through the expression and specificity of the condensing enzyme. *Plant J.* 12 121–131. 10.1046/j.1365-313X.1997.12010121.x 9263455

[B24] Morineau C. Bellec Y. Tellier F. Gissot L. Kelemen Z. Nogue F. (2017). Selective gene dosage by CRISPR-Cas9 genome editing in hexaploid *Camelina sativa*. *Plant Biotechnol. J.* 15 729–739. 10.1111/pbi.12671 27885771 PMC5425392

[B25] Ohlrogge J. Browse J. (1995). Lipid biosynthesis. *Plant Cell* 7 957–970. 10.2307/3870050 7640528 PMC160893

[B26] Okuzaki A. Ogawa T. Koizuka C. Kaneko K. Inaba M. Imamura J. (2018). CRISPR/Cas9-mediated genome editing of the fatty acid desaturase 2 gene in *Brassica napus*. *Plant Physiol. Biochem.* 131 63–69. 10.1016/j.plaphy.2018.04.025 29753601

[B27] Ozseyhan M. E. Kang J. Mu X. Lu C. (2018). Mutagenesis of the FAE1 genes significantly changes fatty acid composition in seeds of *Camelina sativa*. *Plant Physiol. Biochem.* 123 1–7. 10.1016/j.plaphy.2017.11.021 29216494

[B28] Pham A. T. Shannon J. G. Bilyeu K. D. (2012). Combinations of mutant FAD2 and FAD3 genes to produce high oleic acid and low linolenic acid soybean oil. *Theor. Appl. Genet.* 125 503–515. 10.1007/s00122-012-1849-z 22476873

[B29] Qu C. Jia L. Fu F. Zhao H. Lu K. Wei L. (2017). Genome-wide association mapping and Identification of candidate genes for fatty acid composition in *Brassica napus* L. using SNP markers. *BMC Genomics* 18:232. 10.1186/s12864-017-3607-8 28292259 PMC5351109

[B30] Razzaq A. Saleem F. Kanwal M. Mustafa G. Yousaf S. Imran Arshad H. M. (2019). Modern trends in plant genome editing: an inclusive review of the CRISPR/Cas9 toolbox. *Int. J. Mol. Sci.* 20:4045. 10.3390/ijms20164045 31430902 PMC6720679

[B31] Stefansson B. R. Hougen F. W. (1964). Selection of rape plants (*Brassica napus*) with seed oil practically free from erucic acid. *Can. J. Plant Sci.* 44 359–364. 10.4141/cjps64-069

[B32] Stefansson B. R. Hougen F. W. Downey R. K. (1961). Note on the isolation of rape plants with seed oil free from erucic acid. *Can. J. Plant Sci.* 41 218–219. 10.4141/cjps61-028

[B33] Tang S. Zhao H. Lu S. Yu L. Zhang G. Zhang Y. (2021). Genome- and transcriptome-wide association studies provide insights into the genetic basis of natural variation of seed oil content in *Brassica napus*. *Mol. Plant* 14 470–487. 10.1016/j.molp.2020.12.003 33309900

[B34] Taylor D. C. Zhang Y. Kumar A. Francis T. Giblin E. M. Barton D. L. (2009). Molecular modification of triacylglycerol accumulation by over-expression of DGAT1 to produce canola with increased seed oil content under field conditions. *Botany* 87 533–543. 10.1139/B08-101

[B35] Wang N. Wang Y. Tian F. King G. J. Zhang C. Long Y. (2008). A functional genomics resource for *Brassica napus*: development of an EMS mutagenized population and discovery of FAE1 point mutations by TILLING. *New Phytol.* 180 751–765. 10.1111/j.1469-8137.2008.02619.x 18811617

[B36] Wu N. Lu Q. Wang P. Zhang Q. Zhang J. Qu J. (2020). Construction and analysis of GmFAD2-1A and GmFAD2-2A soybean fatty acid desaturase mutants based on CRISPR/Cas9 technology. *Int. J. Mol. Sci.* 21:1104. 10.3390/ijms21031104 32046096 PMC7037799

[B37] Xing H. L. Dong L. Wang Z. P. Zhang H. Y. Han C. Y. Liu B. (2014). A CRISPR/Cas9 toolkit for multiplex genome editing in plants. *BMC Plant Biol.* 14:327. 10.1186/s12870-014-0327-y 25432517 PMC4262988

[B38] Yu N. I. Zhang F. C. Wang Y. C. Fei P. U. Jia-Na L. I. (2011). Cloning and functional analysis of enoyl-CoA reductase gene BnECR from oilseed rape (*Brassica napus* L.). *Acta Agron. Sin.* 37 424–432. 10.1016/S1875-2780(11)60012-6

[B39] Yuan M. Zhu J. Gong L. He L. Lee C. Han S. (2019). Mutagenesis of FAD2 genes in peanut with CRISPR/Cas9 based gene editing. *BMC Biotechnol.* 19:24. 10.1186/s12896-019-0516-8 31035982 PMC6489235

[B40] Zhang D. Zhang Z. Unver T. Zhang B. (2021). CRISPR/Cas: a powerful tool for gene function study and crop improvement. *J. Adv. Res.* 29 207–221. 10.1016/j.jare.2020.10.003 33842017 PMC8020163

