# Supplementary material for: CRISPR/Cas9-Targeted Mutagenesis of BnaFAE1 Genes Confers Low-Erucic Acid in Brassica napus
Source: Front Plant Sci. 2022 Feb 10;13:848723. doi: 10.3389/fpls.2022.848723 (PMC8866690; doi:10.3389/fpls.2022.848723)
Supplement: Supplementary file 2 [file Presentation_1.PPTX]

## Slide 1
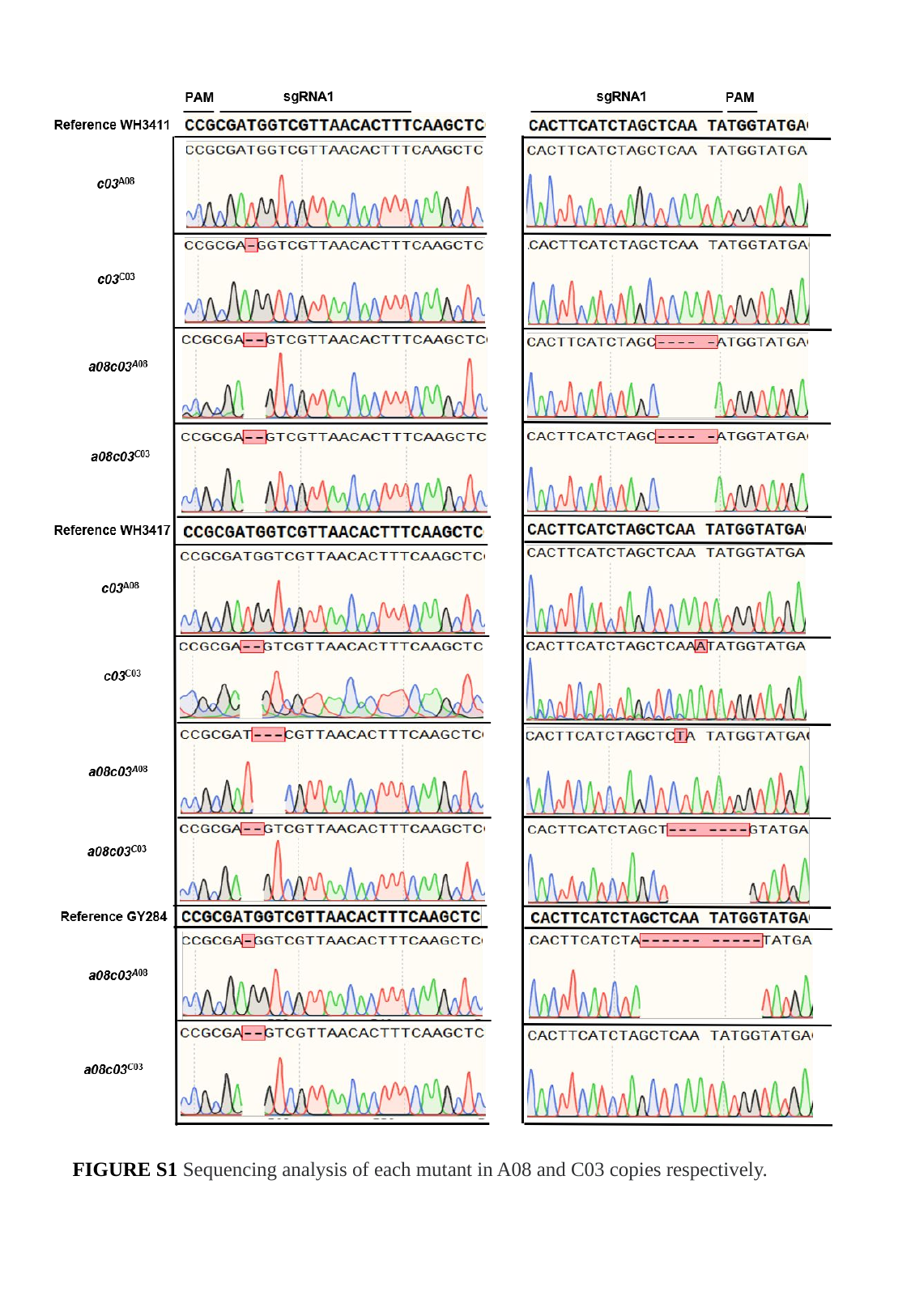

FIGURE S1 Sequencing analysis of each mutant in A08 and C03 copies respectively.

## Slide 2
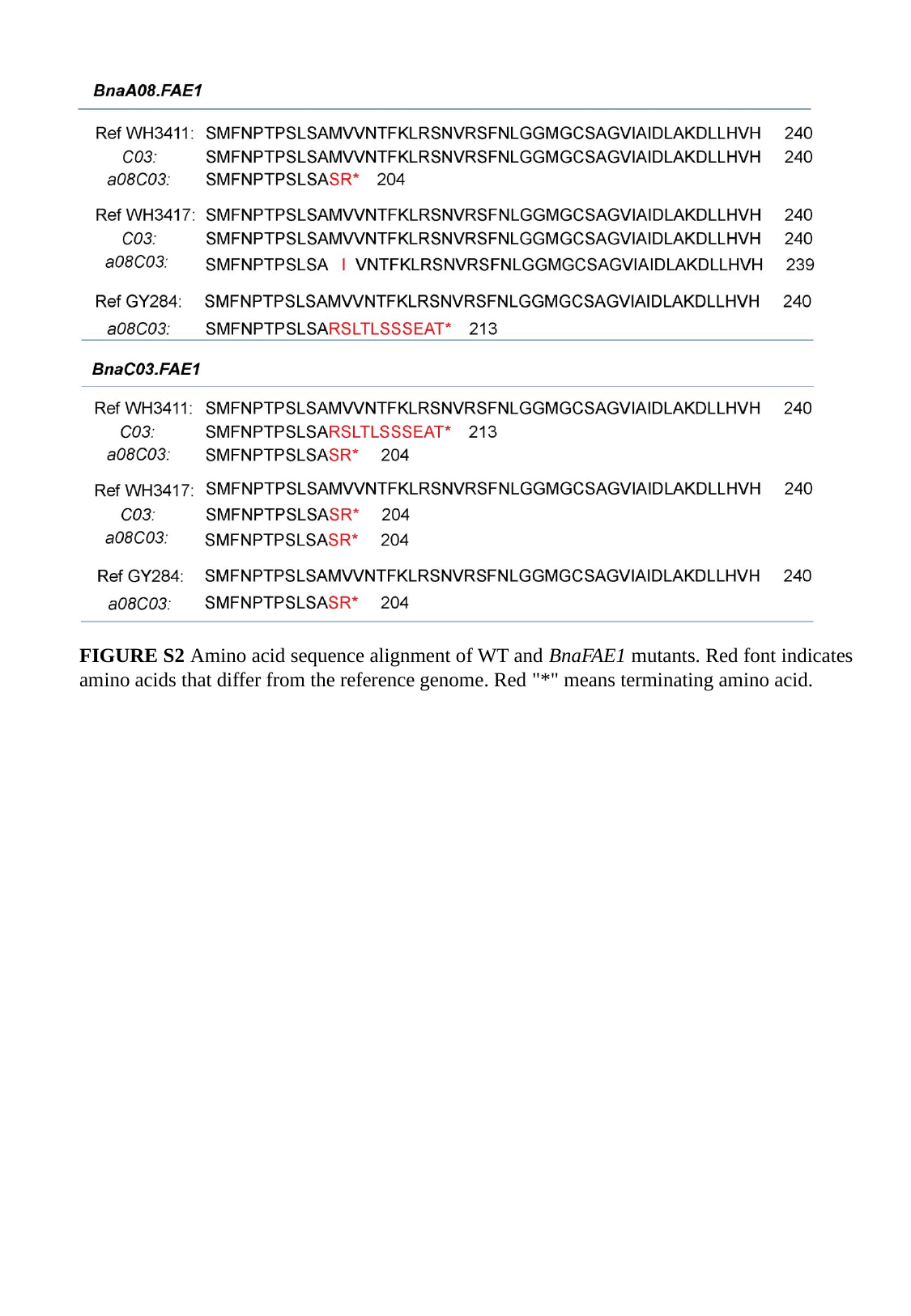

FIGURE S2 Amino acid sequence alignment of WT and BnaFAE1 mutants. Red font indicates amino acids that differ from the reference genome. Red "*" means terminating amino acid.
